# Botulinum Toxin: An Unconventional Tool for the Treatment of Depression?

**DOI:** 10.3390/brainsci15090971

**Published:** 2025-09-10

**Authors:** Matteo Gambini, Riccardo Gurrieri, Gerardo Russomanno, Gianmatteo Cecchini, Federico Mucci, Manuel Glauco Carbone, Donatella Marazziti

**Affiliations:** 1 Department of Clinical and Experimental Medicine, Section of Psychiatry, University of Pisa, 56100 Pisa, Italy; gambinimatteo1996@gmail.com (M.G.); riccardogurrieri4@gmail.com (R.G.); g.russomanno93@gmail.com (G.R.); 2 Medical Center Le Cascine, 56122 Pisa, Italy; cecchini@drcecchini.it; 3 Department of Psychiatry, Lucca Zone, Azienda USL Toscana Nord Ovest, 55100 Lucca, Italy; 4 Department of Medicine and Surgery, Division of Psychiatry, University of Insubria, 21100 Varese, Italy; manuelglaucocarbone@gmail.com

**Keywords:** major depressive disorder, treatment-resistant depression, innovative therapeutic targets, unconventional treatment options, botulinum neurotoxin type A

## Abstract

**Background/Objectives:** Major depressive disorder (MDD) represents a leading cause of global disability, with approximately one-third of patients exhibiting treatment resistance (TRD) despite adequate pharmacological interventions. This treatment gap underscores the urgent need for novel therapeutic strategies. Recently, a series of data suggests that botulinum neurotoxin of type A (BoNT-A), traditionally used for neuromuscular and cosmetic indications, could constitute a potential antidepressant tool. This narrative review critically examines the current preclinical and clinical findings of BoNT-A in MDD. **Methods:** A comprehensive search of PubMed, Scopus, and Web of Science was conducted up to June 2025, including randomized controlled trials, observational studies, animal models, and mechanistic investigations. Search terms included “Botulinum Toxin,” “BoNT type A”, “Depression”, “Major Depressive Disorder”, “Facial Feedback”, and “Neurobiology”. **Results:** Some randomized and observational studies would indicate that glabellar BoNT-A injections might lead to significant reductions in depressive symptoms in patients with MDD and TRD. Proposed mechanisms include both peripheral modulation of emotional expression and brain effects, such as reduced amygdala hyperactivity, increased BDNF expression, and enhanced monoaminergic transmission. Preclinical studies confirm that BoNT-A modulates limbic and brainstem circuits, possibly implicated in affective regulation. The few comparative studies suggest therapeutic efficacy comparable to that of SSRIs, with a more rapid onset. Preliminary data also support its application in bipolar depression and comorbid anxiety disorders. **Conclusions**: The available literature would indicate that BoNT-A might constitute a promising candidate at least as an adjunctive treatment in MDD, although the impact of current findings is limited due to the methodological heterogeneity and the small sample sizes of patients examined. Further large-scale, placebo-controlled trials are warranted to elucidate the mode of action of BoNT-A and to validate or not its clinical effectiveness.

## 1. Introduction

Major depressive disorder (MDD) is a highly prevalent and disabling psychiatric condition. According to the World Health Organization (WHO), more than 264 million people worldwide were affected by depression in 2020, making it a leading cause of disability and one of the major contributors to the global burden of disease [1,2]. This condition is particularly relevant amongst women who are approximately twice as likely as men to suffer from MDD [3,4,5,6]. Again, MDD is associated with an increased risk for developing other psychopathological disorders, and also with a broad range of medical/internal diseases. Regarding specific conditions, the most robust data and with the largest effect size concern heart disease, stroke, diabetes mellitus, headache, asthma, and pain syndromes [6,7,8,9,10,11,12,13].

Although the precise neurobiological mechanisms underlying MDD remain unclear, several converging lines of evidence implicate dysfunctions in the intertwined monoaminergic transmission, immune system, and neuroplasticity [7,8,9]. According to the classical monoamine hypothesis, depression is associated with a reduced availability of serotonin (5-HT), dopamine (DA), and noradrenaline (NE), which, respectively, modulate mood regulation, reward processing, and emotional arousal [10,11]. The decreased activity of these neurotransmitters may result from altered synthesis, transport, receptor sensitivity, enzymatic degradation (e.g., via monoamine oxidase), and signal transduction pathways [12,13]. These neurochemical alterations are thought to contribute to structural and functional brain changes due to altered neuroplasticity observed in MDD, including reduced volumes of the hippocampus, prefrontal cortex (PFC), anterior cingulate cortex (ACC), ventral striatum, and insula, alongside amygdala abnormalities showing a biphasic pattern of enlargement in early stages followed by shrinkage in chronic phases [14,15,16]. Contributing biological factors include chronic immune–inflammatory activation, hypothalamic–pituitary–adrenal (HPA) axis hyperactivity, and reduced levels of brain-derived neurotrophic factor (BDNF) [17,18,19]. Antidepressant treatment has been shown to positively modulate many of these biological processes and to promote neuroplasticity with a partial structural recovery of affected brain regions [20,21,22,23,24]. The current therapeutic guidelines indicate that selective serotonin reuptake inhibitors (SSRIs) are the first-line treatment for MDD [25,26,27,28]. In case of no response, the next strategy is to increase the dose of the prescribed drug. However, the evidence on this type of approach is not so robust, so that the switch to another compound and add-on strategies represents a better approach in terms of efficacy in the literature [29]. The switch, which consists of replacing the active drug used with one belonging to the same or another pharmacological class, has the advantage that, by using a smaller number of drugs, the risk for side effects is reduced, and by improving the patient’s compliance. The add-on strategy that is the addition of another molecule, generally belonging to a class of drugs with a different mechanism of action, would reduce the latency period necessary to obtain a clinical response [27,28]. In case of failure of SSRIs at the optimal dose for at least four to six weeks, the main English and American guidelines on treatment-resistant depression (TRD) suggest preferring, where possible, to wait further for another two to four weeks. In case of no response, first switch to another SSRI, or duloxetine, venlafaxine, mirtazapine, bupropion, and second to tricyclics (TCAs) or monoamine oxidase inhibitors (MAOIs). In case of failure to respond to the above interventions, or partial or need for more rapid response, proceed with the add-on with lithium salts, and then with quetiapine, aripiprazole, mirtazapine, or mianserin. The next steps include T3, combined olanzapine/fluoxetine, risperidone, or another antidepressant, and lately dopamine agonists (pramipexole, ropinirole), psychostimulants, buspirone or pindolol, anticonvulsants, omega-3 fatty acids, or folates [27,28].

Nevertheless, a significant proportion of patients (about one third) fail to achieve adequate symptom remission and become treatment resistant [20]. The main problem in dealing with TRD is the lack of an univocal definition. Currently, the most widely accepted definition is identifying patients as resistant when not responding to at least two different antidepressant treatments administered at the appropriate dose and for an adequate duration [21]. However, according to some authors, this definition is too limiting, as it does not take into account partial responses or add-on therapeutic options, as well as the class of drugs used and their mechanism of action [30,31,32,33]. To better understand the extent of the problem, a survey conducted in the United States estimated TRD in terms of both health and public spending on pharmacologically treated MDD. The results showed that the 12-month prevalence of pharmacologically treated MDD involved 8.9 million adults, of whom 2.8 million (30.9%) had TRD [23]. The total annual cost of pharmacologically treated MDD was $92.7 billion, of which $43.8 billion (47.2%) attributable to TRD [23]. Another element to take into consideration in the debate on TRD is the so-called “pseudo-resistance” [24]. It consists of an inadequate response to treatment due to one or more of the following factors to be taken into consideration: administration of drugs at inadequate doses and times, therapeutic non-compliance, wrong differential diagnosis, psychiatric and general medical comorbidities, medical therapies (which can modify the pharmacokinetics and pharmacodynamics of the psychotropic drugs used), socio-demographic, clinical, and biological factors [34,35].

Since data on TRD are rather scarce and controversial, this article aimed to review the existing literature on new treatment options for MDD and especially for TRD, including the potential use of facial injections of botulinum toxin (BoNT), with a particular attention to its mode of action, efficacy, and safety profile.

## 2. Methods

**Figure 1:**
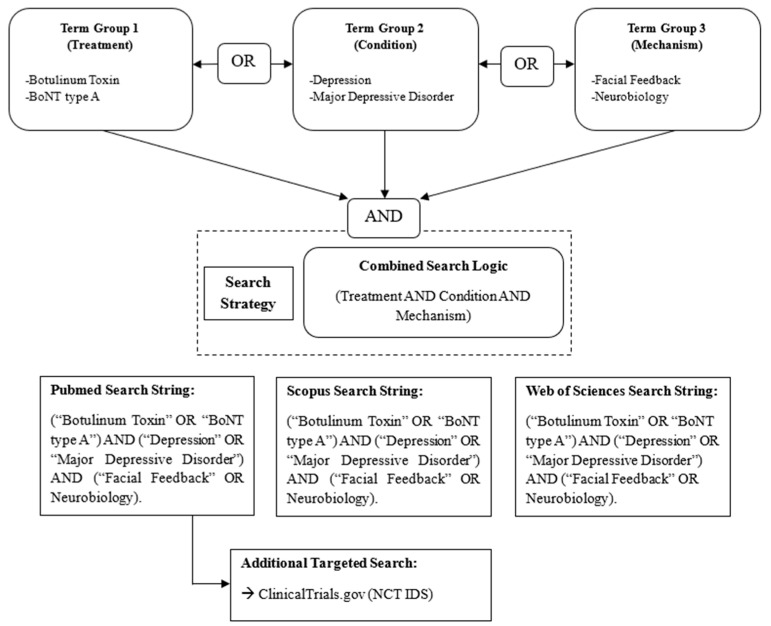
Conceptual Networking Diagram of Literature Search Strategy.

The present paper is a narrative literature review aiming to summarize and critically appraise the available evidence on the use of BoNT in the treatment of MDD. Both preclinical and clinical studies were included. A comprehensive literature search was performed in PubMed, Scopus, and Web of Science databases from the first reports on botulinum toxin in psychiatry up to June 2025, using combinations of the following terms: “Botulinum Toxin”, “BoNT type A”, “Depression”, “Major Depressive Disorder”, “Facial Feedback”, and “Neurobiology” (Figure 1). Inclusion criteria were as follows: Peer-reviewed original studies (randomized controlled trials, observational studies, preclinical models, mechanistic studies) addressing the use of botulinum toxin in depressive disorders; Articles in English; Studies explicitly reporting clinical or neurobiological outcomes relevant to depression. Exclusion criteria were as follows: Non-peer-reviewed sources; Studies not involving depression or not directly related to BoNT-A. Study selection was performed by the authors based on the coherence of the study with the aims of the review (initial screening by title/abstract, followed by full-text evaluation). Given the narrative nature of this review, no formal quality assessment or meta-analysis was performed.

## 3. Results

### 3.1. Novel Treatment Options for TRD

In the following section, we shall provide a brief overview of the latest emerging treatment strategies for MDD acting through mechanisms beyond the traditional monoaminergic paradigm. Subsequently, particular attention will be devoted to the potential role of botulinum toxin in MDD and TRD.

#### 3.1.1. Promising Experimental Drugs for TRD

Within the sprawling nosology of TRD wherein pharmacological nonresponse often masks a mosaic of immune–metabolic, glutamatergic, or endocrine derangements rather than mere receptor desensitization, an emergent corpus of trials seeks to operationalize “precision psychiatry” by prospectively stratifying patients along inflammatory gradients, metabolic phenotypes, or rapid-acting neuromodulatory pathways. The fourteen ClinicalTrials.gov protocols collated here range from quadruple-blind Phase 3 add-on designs embedding dense cytokine and kynurenine readouts to single-arm feasibility pilots geared toward effect-size estimation, while uniformly contending with modest sample sizes, abbreviated follow-up windows, or open-label architectures that circumscribe external validity even as they illuminate mechanistic plausibility (e.g., Magnetic Resonance-Spectroscopy γ-aminobutyric acid shifts, high-frequency cognitive sampling) (Table 1).

Leveraging the observation that approximately one-third of depressed individuals exhibit low-grade inflammation, the INSTA-MD trial randomizes non-remitting DSM-5 MDD patients—prospectively stratified by high-sensitivity C-Reactive Protein (CRP; <3 vs. >3 mg/L)—to adjunctive minocycline (100 mg twice daily) or celecoxib (200 mg twice daily) versus placebo, each atop treatment as usual (TAU), designating both Hamilton Depression Rating Scale-17 change and remission (≤7) at 12 weeks as co-primary endpoints, while deploying an unusually comprehensive secondary battery spanning Inventory of Depressive Symptomatology Self-Report (IDS-SR) trajectories, psychomotor disturbance (Clinical Outcomes in Routine Evaluation, CORE), sleep (Pittsburgh Sleep Quality Index, PSQI), anxiety (State-Trait Anxiety Inventory, STAI), adherence (Medication Adherence Report Scale, MARS), and a multiplex immune–metabolic and kynurenine panel—a six-arm, biomarker-anchored architecture that is methodologically audacious yet inevitably vulnerable to attrition and multiplicity effects (NCT05644301). Conceptually consonant, the INFLAMED study constrains eligibility to an immune–metabolic depression phenotype-defined by atypical, energy-related symptoms (IDS—Anergia Subscale ≥ 6) together with CRP > 1 mg/L-and tests celecoxib 400 mg/day as add-on to SSRI or Serotonin-Norepinephrine Reuptake Inhibitor (SNRI)-based TAU versus placebo over 12 weeks, modeling IDS-SR symptom trajectories bi-weekly and enumerating response, remission, fatigue, craving, sleep, disability and metabolic–inflammatory indices, thereby maximizing internal coherence at the expense of broad generalizability (NCT05415397). Pushing further down the cytokine cascade, a Phase 2 mechanistic RCT administers a single 5 mg/kg infliximab infusion to 25–50-year-old patients with CRP ≥ 3 mg/L and moderate depressive severity, interrogating psychomotor speed and executive function through daily, smartphone-based cognitive probes over two weeks, an elegant, high-granularity sampling scheme whose temporal brevity limits inference on durability (NCT06136546). Finally, the CODA feasibility study, open-label and single-arm by design, recruits obese (Body Mass Index ≥ 30 kg/m^2^) TRD patients with CRP ≥ 3 mg/L to 8 weeks of adjunctive minocycline 200 mg/day, prioritizing enrolment and adherence metrics, completeness of biomarker and Magnetic Resonance Imaging (MRI) acquisitions, and preliminary effect-size calculations, explicitly positioning itself as a scaffold for a fully powered randomized trial (NCT06537921).

Pivoting from overt immunomodulation to interventions that recalibrate metabolic homeostasis or steroidogenic/endocrine signaling, several studies interrogate whether correcting dysregulated energy utilization, glucose handling, lipid profiles, or mineralocorticoid tone can secondarily ameliorate depressive phenomenology. An open-label, single-arm feasibility trial of a rigorously dietitian-supervised ketogenic diet, two weeks of induction followed by ten weeks of maintenance, explicitly adjunctive to first-line SSRIs, prioritizes adherence as its primary endpoint while serially sampling anhedonia (Snaith-Hamilton Pleasure Scale, SHAPS), reward motivation (Effort Expenditure for Rewards Task, EEfRT), depressive severity (Montgomery-Åsberg Depression Rating Scale, MADRS), and an extensive panel of inflammatory and neurotrophic biomarkers (BDNF, TNF-α, IL-1, IL-6, IL-10), thereby trading internal controls for granular mechanistic readouts in a deliberately small cohort (*n* = 15) (NCT05558995). In a conceptually aligned yet pharmacological approach, a Phase 2, six-week, single-group study administers the sodium–glucose cotransporter 2 (SGLT2) inhibitor empagliflozin (10 mg for two weeks then 25 mg for four), targeting adults with moderate MDD (MADRS ≥ 20) and ≤ 2 prior adequate antidepressant failures, and tracks changes in MADRS, suicidality (Columbia-Suicide Severity Rating Scale, C-SSRS), and anhedonia (SHAPS), a design whose open-label architecture and tiny sample (*n* = 16) nonetheless constrain causal inference (NCT05757791). By contrast, the statin trial randomizes mildly-to-moderately depressed adults (20–45 years) to rosuvastatin 10 mg/day for 12 weeks versus sertraline, anchoring efficacy in MADRS change and monitoring lipid parameters, an elegant repurposing premise hampered, however, by absent blinding information and an unclear TRD focus (NCT06698666). Endocrine modulation is pursued more directly in a double-blind, placebo-controlled study of enoxolone, a peripheral 11β-hydroxysteroid dehydrogenase type 2 (11β-HSD2) inhibitor that enhances cortisol access to mineralocorticoid receptors and suppresses Renin–Angiotensin–Aldosterone System (RAAS) output; here, patients are stratified by systolic blood pressure and overnight aldosterone/cortisol ratios, with exploratory splits on sleep duration, heart-rate variability, salt preference, C-reactive protein, and structural or diffusion tensor neuroimaging, an ambitious biomarker matrix whose clinical endpoint is less explicitly delineated (NCT05570110). Finally, a Phase 2 trial in People Living with Human Immunodeficiency Virus (PLWH) and depression randomizes participants to pregnenolone escalated to 500 mg/day by week 4 and maintained for another 4 weeks or placebo, allowing continuity of antidepressants, and designates left insular cortex GABA concentration (Magnetic resonance spectroscopy, MRS) at days 14 and 56 as the primary outcome while secondarily interrogating Center for Epidemiologic Studies-Depression scale (CES-D) improvement, pro-inflammatory monocyte subsets (CD14^+^CD16^+^), and safety/dose-modification metrics, thereby marrying neurosteroid replacement to neurochemical and immunologic phenotyping but at the cost of population specificity and a relatively small placebo arm (30/120) (NCT05570812).

Extending the paradigm inaugurated by ketamine toward agents that either antagonize the N-methyl-D-aspartate receptor or more broadly perturb glutamatergic and serotonergic signaling, four protocols explore inhaled noble gases or classic tryptamines as ultra-rapid antidepressant or anti-suicidal interventions: a Phase 2, parallel-group trial randomizes outpatients with major depressive disorder to four weekly 60 min sessions of nitrous oxide at either 25% or 50% versus an oxygen–air placebo mixture, anchoring efficacy to Hamilton Depression Rating Scale-21 change over four weeks and embedding dose–response modeling, daily mood profiling (Profile of Mood States, POMS), computerized adaptive assessments of depression, anxiety and suicidality (Computerized Adaptive Test for Mental Health, CAT-MH), and formal suicidality tracking (Sheehan Suicidality Tracking Scale, S-STS), all under double-blind conditions that nonetheless cannot fully obviate the sensory detectability of nitrous oxide (NCT05357040). In a diametrically different clinical context, a second, smaller Phase 2 trial delivers a single 45 min 50% nitrous oxide session-or placebo-plus TAU to acutely suicidal, non-psychotic MDD patients in the emergency department, operationalizing feasibility and safety while designating 24-h CAT-MH shifts in suicidality, depression, and anxiety as the primary endpoint, with rapid (30–60 min) and sustained responses as secondary targets—an elegant proof-of-concept whose external validity hinges on Emergency Department (ED) logistics and stringent exclusion criteria (NCT05710887). Xenon, another inert gas with potent anti-glutamatergic properties, is interrogated in an early Phase 1 randomized, double-blind crossover design in which 20 severely depressed individuals (split evenly between MDD and bipolar depression) inhale xenon–oxygen (35:65) and nitrogen–oxygen (35:65) in counterbalanced order atop TAU, with day-1 improvements on a 6-item HDRS and QIDS-C, plus densely spaced post-administration ratings (40–230 min; days 1, 3, 7), capturing the kinetics of response yet sacrificing long-term outcomes and scalability (NCT03748446). Finally, a Phase 1 randomized, triple-blind crossover study administers low and medium intravenous bolus/infusion doses of dimethyltryptamine (DMT) and, in separate sessions, Δ9-tetrahydrocannabinol (THC) or placebo to depressed adults (moderate–severe MDD with ≥1 inadequate antidepressant trial) and healthy controls, prioritizing safety (blood pressure, heart rate, oxygen saturation), phenomenology (Mystical Experience Questionnaire, Psychotomimetic States Inventory, Challenging Experience Questionnaire), reinforcement metrics, and electrophysiological correlates (resting-state electroencephalography), while probing expectancy and blinding integrity, thus privileging mechanistic depth over clinical generalizability and extending psychedelic inquiry beyond psilocybin-centric paradigms (NCT06671977).

A distinct, non-pharmacological vector is represented by whole body hyperthermia delivered via water-filtered infrared radiation in a rigorously quadruple-blind, sham-controlled, six-week trial that randomizes German-speaking adults with DSM-5 MDD (Hamilton Depression Scale, HAMD-17 ≥ 14) to active versus sham heating, positing observer-rated HAMD-17 change at one week as the primary endpoint and tracking self-reported depressive symptoms (Beck Depression Inventory, BDI) and quality of life (Medical Outcomes Study Short Form, MOS-SF) thereafter; while conceptually attractive in its bid to harness immune–metabolic and autonomic shifts induced by controlled hyperthermia, the modest planned sample (*n* = 30) and the short primary window inevitably circumscribe the statistical power to detect clinically meaningful differences (NCT06323785).

Taken together, these heterogeneous yet thematically convergent trials instantiate a translational shift from monolithic monoaminergic augmentation toward stratified, mechanism-informed interventions, whether by dampening cytokine cascades in immuno-metabolic subtypes, reprogramming cellular energetics and lipid metabolism, modulating mineralocorticoid signaling, or triggering rapid synaptic recalibration through anti-glutamatergic or psychedelic agents, while also experimenting with somatic paradigms that exploit systemic physiological perturbation; however, the field remains constrained by small, often single-site samples, open-label or single-arm designs, short follow-up horizons, and, not infrequently, co-primary endpoints and biomarker multiplicity that raise issues of type-I error and reproducibility, underscoring the imperative for larger, multisite, adaptive trials that validate predictive biomarkers and embed longer-term functional outcomes before any of these “non-conventional” avenues can be responsibly assimilated into routine TRD algorithms.

#### 3.1.2. Botulinum Neurotoxin

Currently, the botulinum neurotoxin type A (BoNT-A) is successfully used in the management of a variety of medical conditions, particularly for the symptomatic relief of blepharospasm, cervical dystonia, sialorrhea, different focal muscle spasticity, and for the temporary improvement of dynamic facial wrinkles [30,31,32]. It is a zinc-dependent endopeptidase composed of a light chain (50 kDa) and a heavy chain (100 kDa) linked by disulfide bonds [33]. Botulinum toxins inhibit the release of acetylcholine into the synaptic cleft and thus cause temporary muscle paralysis [36]. Botulinum toxins are produced by several strains of *Clostridium botulinum* and are classified into seven serotypes from A to G [37]. Botulin neurotoxin type A (BoNT-A) and BoNT-E cleave the synaptosome-associated protein of 25 kDa (SNAP-25) [38], while BoNT-B, -D, -F, and -G cleave the vesicle-associated membrane protein (VAMP), also known as synaptobrevin [39]. The FDA and EMA approved three BoNT-A products and one BoNT-B for therapeutic use, with BoNT-A being the most prescribed [40]. This is due to the high potency of BoNT-A, which makes it between 3 and 6 months, thanks to the restoration of the turnover of the Soluble N-ethylmaleimide-sensitive factor attachment protein receptor (SNARE) protein complex [33]. The safety profile is good, with a reduced risk of side effects in both the acute phase and sensitization episodes with the production of autoantibodies by the patient’s immune system [41].

Specifically, the BoNT-A is a metalloprotease that, when injected locally, exerts its action at the neuromuscular junction by cleaving the SNAP-25, a SNARE, essential for the fusion of synaptic vesicles with the inner surface of the cell membrane, producing a prolonged, albeit transient block of neurotransmitter release from peripheral nerve endings and thus inducing muscle relaxation [42,43]. Since not all the observed clinical effects of BoNT-A can be explained on the basis of its peripheral actions, cellular and animal models highlighted that there could be a retrograde transport of the catalytically active toxin in projection neurons. This long-range transport process is followed by transcytosis and action on second-order synapses [44]. In any case, the few data in humans highlight a reduction in inhibition mediated by the Renshaw cells [44]. Therefore, it was hypothesized that the combination of both peripheral and central effects could explain not only the already well-known beneficial effects in the fields of esthetic/cosmetic medicine, but also its potential use for therapeutic purposes in psychiatric disorders, particularly in MDD, but maybe not only.

Although not yet approved for its use in depression, an increasing body of evidence underlines that BoNT-A injection in the glabellar area may show beneficial effects in patients with TRD [45]. The main advantages of BoNT-A are the following: a good degree of efficacy in terms of effect size associated with an acceptable safety profile, mild adverse reactions, long-term effects with a single injection, as well as an extremely low risk of abuse or addiction [46,47]. Conversely, the limitations are the lack of precise knowledge of the mechanism of action correlating with the variability in individual response and the efficacy, mainly documented in patients with moderate forms of depression [48].

### 3.2. Neurobiological Mechanism of Action of BoNT-A

Several authors proposed that the antidepressant effect of BoNT-A may, at least in part, involve the retrograde transport to the central nervous system (CNS) [42,49,50], although systemic levels are supposed to remain extremely low [51].

Two non-mutually exclusive mechanisms have been hypothesized to account for its central effects [50]. The first involves direct retrograde transport of BoNT-A to the cerebral cortex via motor neurons, followed by transcytosis to second-order neurons [50]. While only trace amounts reach the brain, insufficient to cause cytotoxic effects, this pathway may still be sufficient to produce therapeutic neuromodulation [49]. Specifically, this mechanism has been linked to reduced inhibition mediated by Renshaw cells, potentially contributing to the antidepressant response [49].

The second mechanism posits that BoNT-A induces neuroplastic change secondary to peripheral neuromuscular blockade. Specifically, the lack of use of a body part following BoNT-A could decrease its representation in the brain and increase the representations of other nearby ones [50]. The BoNT-A would, therefore, act by reducing the cortical representation of the injected muscles, since there will be less afferent activity from them, and this could help to normalize their function [50].

Support for these hypotheses derives from preclinical models. In one study, mice subjected to stress-induced depression paradigms (e.g., spatial restriction stress, forced swim test, tail suspension test, sucrose preference test, open-field test) showed significant reductions in depressive-like behaviors and of altered neurochemical parameters following a single BoNT-A injection [52]. The BoNT-A led to a significant increase in 5-HT levels in the hippocampus and hypothalamus, in the expression of the N-methyl-D-aspartate (NMDA) receptor NR1 and NR2B subunits in the hippocampus, and in the BDNF of the hippocampus, hypothalamus, prefrontal cortex, and amygdala [52]. Finally, BoNT-A transiently increased the levels of phosphorylated extracellular signal-regulated kinase (p-ERK) and cAMP response element-binding protein (p-CREB) [52].

A more recent study proposed an additional mechanism involving axonal transport from facial muscles to central motor pathways. Mice subjected to chronic restraint stress (CRS), another validated model for inducing depression-like behavior, received BoNT-A injections into the unilateral intrinsic whisker musculature (WIM), and behavioral testing confirmed a reduction in these behaviors [53]. Mice were then sacrificed for immunostaining studies showing that axonal retrograde administration of BoNT-A to the soma of whisker-innervating facial motor neurons (wFMN) and subsequent transcytosis to the synaptic terminals of second-order neurons induced central effects [53]. It was also observed that CRS-induced expression of c-Fos and CaMKII double-positive neurons in the ventrolateral periaqueductal gray (vlPAG), which send afferents to wFMN, was downregulated 3 weeks after facial BoNT-A administration [53]. The evidence from preclinical studies is summarized in Table 2. 

**Figure 2:**
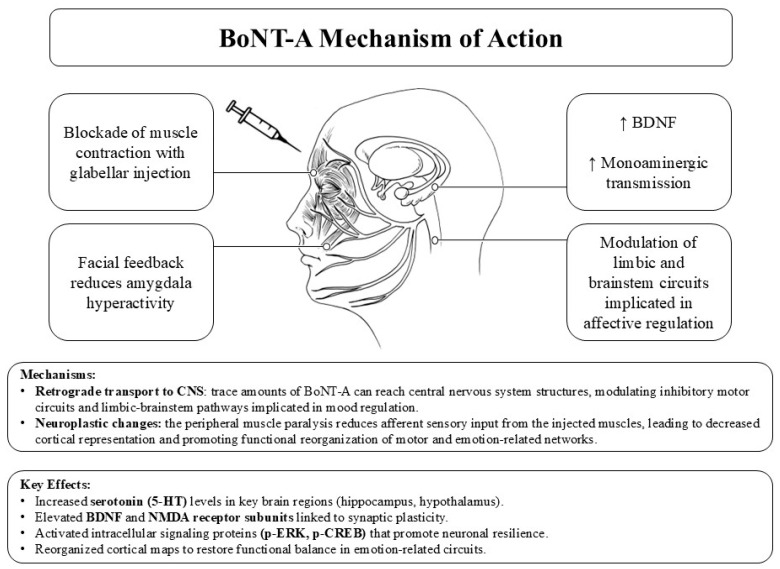
BoNT-A Mechanism of Action. The upward arrows (↑) indicate an increase.

Taken together, these data suggest that facial BoNT-A administration could modulate limbic–brainstem circuits involved in affective regulation (Figure 2). Rather than providing a competing model, this mechanism may complement the previously described pathways, contributing to a more integrative understanding of BoNT-A effects in the CNS.

### 3.3. Why Botulinum Toxin May Be Effective in Depression

Some hypotheses have been proposed to explain the antidepressant effects observed following the local administration of the BoNT-A in human beings. The first, called the facial feedback hypothesis, derives from the observation that contraction of frown lines is associated with negative emotional states [56], and that depressed individuals tend to exhibit increased facial muscle activity compared to healthy individuals [57,58]. According to the proposers, facial expressions would provide proprioceptive feedback to the brain, while reinforcing the emotional states they represent. By relaxing the corrugator and procerus muscles through BoNT-A injections, this feedback loop is interrupted and might lead to a reduction in negative affect and an enhancement of positive emotional states [59,60]. In 1872, Charles Darwin supposed that facial activity might influence mammalian and human emotional responses [61], as successfully confirmed after more than one century [62]. Historical practices also lend indirect support to this model. However, electroacupuncture at the “YingTang” point, corresponding anatomically to the glabellar region, has long been used in ancient times to alleviate depressive-like conditions [63]. Moreover, a stronger antidepressant response to BoNT-A has been reported in individuals with higher baseline agitation, potentially due to greater psychomotor facial activity [64]. Contemporary findings further support a bidirectional signaling pathway between facial musculature and the emotional centers of the brain [65]. Emotional proprioception, a mechanism by which facial muscle activity informs the brain of emotional state, is believed to play a key role [66,67]. Through the process of facial embodiment, even subtle emotional cues can become fully integrated into felt experience [68]. Relaxation of the glabellar region not only softens facial expression but may directly attenuate negative affect by disrupting these feedback loops [59]. In addition to its purely esthetic benefits, the treatment, as applied in esthetic medicine, appears to improve emotional wellbeing, social and psychological behavior, and reduce irritability, as well as depressive and anxious moods [69,70,71,72].

Beyond the peripheral mechanism, BoNT-A appears to influence central emotional processing. As already mentioned, neuroimaging studies reported reduced activation of the amygdala, a structure implicated in fear, anger, and depressive mood, following BoNT-A injection in the glabellar area [69,73,74,75,76,77]. Injection of BoNT-A into the corrugator muscle may block normal sensory feedback from nerves, particularly the left amygdala, to the brain [78]. It has been observed that frontal palsy secondary to the effect of the administration of the neurotoxin, triggers a signal to the proprioceptive fibers of the optic branch of the trigeminal nerve, which in turn is transmitted to the mesencephalic nucleus and finally to the amygdala, which connects to the ventromedial prefrontal cortex [79], both systems involved in the regulation of current patterns of depression [80]. It is known that an altered functioning of the amygdala–ventromedial prefrontal cortex pathway has been reported in MDD, together with hyperactivation of the amygdala [76,77,81]. This neuro-functional correlation has been associated with negative emotions such as anger, depression, anxiety, and fear. However, the BoNT-A, by reducing its degree of activation by blocking the release of acetylcholine [54], would improve mood [46]. Some researchers tested the response of the amygdala after a period sufficient for the effect of BoNT-A on muscle contraction to completely wear off, while reporting that its activity had returned to its original state [76,77]. These results confirmed that BoNT-A may reversibly reduce amygdala activity [77]. Indeed, the amygdala, through its connections with the hypothalamus and brainstem regions, can modulate different autonomic functions, including blood pressure, heart rate, and respiration [82,83]. By converting emotional and sensory information into coordinated autonomic responses, it is able to create a connection between affective states and somatic responses [84,85,86]. Alterations in amygdala signaling can also cause autonomic imbalance, resulting in maladaptive sympathetic or parasympathetic responses, as observed in various neuropsychiatric disorders [87,88,89]. Restoring normal amygdala activity would improve, in this way, not only the emotional and behavioral regulation, but also autonomic responses [90,91,92].

In addition to neurobiological mechanisms, other mechanisms have been hypothesized that could play a role in alleviating depressive symptoms [93]. Reducing frowning can alter self-perception and how others respond, potentially improving mood through enhanced social feedback and interpersonal interactions [94]. These changes may promote positive emotions and reduce social withdrawal, which are common in depression. Several studies have reported improved quality of life, reduced fear and sadness, and enhanced emotional wellbeing following BoNT-A treatment, even when administered for esthetic purposes [59,70,71,95].

Taken together, BoNT-A appears to exert antidepressant effects through a multifaceted mechanism that includes disruption of negative facial feedback, modulation of limbic circuits, and enhancement of self-perception and social interactions. This integrative model supports the growing interest in BoNT-A as a novel intervention for mood disorders.

### 3.4. Botulin Toxin in the Treatment of MDD: Clinical Evidence

The first observation supporting the potential antidepressant effects of BoNT-A emerged in 2006, with a pilot study reporting depressive symptom improvement after glabellar injections [96]. Since then, some RCTs, although still involving a limited number of patients, replicated and expanded these findings, also showing that the benefits persisted for several months [97,98,99].

A more recent phase II randomized, double-blind, placebo-controlled trial conducted exclusively on female subjects examined two BoNT-A doses, specifically, 30 units (U) and 50 U, and revealed a greater efficacy and a faster onset of symptom relief with the 30 U dose [100]. The authors hypothesized that the decreased response to the higher dose might be related to increased placebo responsiveness and procedural expectations, so they highlighted the need to deepen the optimal dose and injection pattern [100]. Although this trial involved only women, as already noted, it is interesting that another trial reported no differences between the two sexes [101]. A recent study carried out in 140 patients confirmed no significant gender differences in depression improvement after 50 or 100 U of BoNT-A, as well as the benefit of the lowest dose [102].

A study conducted during the SARS-CoV-2 pandemic was the first to evaluate the effects of BoNT-A administration in habitual users who, due to the restrictions caused by the lockdown, could not follow their usual administration for a minimum of 20 weeks [103]. A total of 45 people were recruited, of whom 30 were assigned to the treatment group, which was supposed to receive between 20 U and 64 U of BoNT-A in the upper third of the face, and the remaining 15 to the control group that included the administration of saline. Both groups were evaluated using the psychometric scales FACE-Q Lines Between Eyebrows [104] and Subjective Happiness Scale (SHS) [105] at baseline and 2 weeks later [103]. After one month, the subjects belonging to the control group were transferred to the treatment group and received the BoNT-A administration and were then re-evaluated after an additional two weeks [103]. This study confirmed the significant mood improvement following treatment with BoNT-A compared to the control group, which did not experience any improvement until it also received the toxin [103]. In addition, the ensuing findings underline that habitual users continued to experience the improvement in mood, as long as they took or resumed the administration [103]. This suggests that the mechanism involved in the beneficial effect exerted on mood is not susceptible to tolerance, and that it maintains its effects substantially unchanged if the treatment goes on.

The magnitude of the efficacy of BoNT-A treatment was also compared to that of sertraline, a first-line SSRI [88]. The study included a total of 76 patients, of whom 52 were treated with 100 U of BoNT-A and the remaining 24 being treated with sertraline at a dose ranging between 50 and 200 mg, on the basis of clinical need. The HAMD-17, the Hamilton Anxiety Scale (HAMA), the Self-rating Depression Scale (SDS), and the Self-rating Anxiety Scale (SAS: https://psychology-tools.com/test/zung-anxiety-scale; the date of access: 7 September 2025) were used to assess severity and type of symptoms at baseline and after 12 weeks [88]. Both groups showed a significant improvement from baseline; however, BoNT-A resulted in even superior to sertraline in terms of efficacy and onset of response (that was detected already after 2 weeks, compared to 3 weeks of sertraline) [88]. A comparative study between BoNT-A and sertraline, conducted on patients with post-stroke depression, confirmed the previous data and the earlier onset of the therapeutic response [106].

To the best of our knowledge, the first randomized, double-blind, placebo-controlled trial was conducted on a sample of 88 patients of Chinese origin, of whom 56 were included in the BoNT-A treatment arm and 22 in the placebo arm [107]. The clinical features were assessed by means of the HAMD and HAMA and the SDS and SAS, at baseline and after a 12-month follow-up [107]. This study highlighted the efficacy of BoNT-A on both anxious and depressive symptoms, with a more robust effect on the former.

A recent retrospective study included 51 outpatients, mostly seeking treatment for depression, who had received at least one BoNT-A administration to the glabellar region. Some of them had also received injections in other parts of the face (the mouth), others multiple treatment sessions. Among the enrolled patients, 50% had comorbidity with other psychiatric conditions, mainly generalized anxiety disorder (GAD) [108] and borderline personality disorder (BPD) [108,109,110]. The results were interesting, as it was evident that BoNT-A improved all psychiatric symptoms, especially the depressive ones [108]. No loss of efficacy was observed following repeated administrations of the treatment [108]. Injections in other facial areas produced the same effects as the glabellar administration [108,111].

In a recent retrospective study, the authors tested the therapeutic outcome of BoNT-A administration in regular users compared to treatment-naive subjects [112]. A total of 100 patients of both sexes were enrolled; inclusion criteria required 50 of them to be regular users (at least one administration per year for at least five consecutive years) and 50 who had never undergone this type of therapy. They were assessed by means of the BDI and Beck Anxiety Inventory (BAI) [112]. A significant reduction in depression and anxiety levels was observed in subjects who regularly received neurotoxin injections compared to those who had never received them [112].

For the sake of completeness, we mention that the evidence regarding the use of BoNT-A in bipolar depression remains extremely limited and inconclusive, primarily due to the small number of available case reports. In the first study, the only participant with a diagnosis of bipolar disorder exhibited only partial symptomatic improvement, although some enhancement was observed on psychometric scale scores [96]. More promising results emerged from a subsequent case series in which six patients with bipolar disorder experiencing moderate to severe depressive episodes received BoNT-A injections into the frontalis muscles. Their symptoms were evaluated using the BDI-I and II, the MADRS, and the Quick Inventory of Depressive Symptomatology–Self-Report (QIDS-SR16) [113]. Four of the six patients achieved complete symptomatic remission at follow-up, while the remaining two demonstrated substantial clinical improvement. Although symptoms tended to recur after the effects of BoNT-A wore off, re-administration consistently restored the therapeutic response [113]. More recently, a further case series provided additional support for the potential efficacy of BoNT-A in bipolar depression, suggesting that its therapeutic benefits may extend beyond unipolar forms of the disorder [114].

Headache and irritation at the point of administration are the most common side effects, but their severity is mostly mild, as well as predominantly local and of short duration [97,98]. A study designed to evaluate the side effects confirmed that it is a generally safe procedure, mostly associated with mild side effects similar to those of a placebo [100]. Less frequent adverse reactions, occurring in just over 5% of participants, included upper respiratory tract infections and ptosis; the latter was more common in the BoNT-A group, but remained mild and promptly resolved after treatment discontinuation [100]. Gastrointestinal side effects were rare and comparable between the treatment and placebo groups. Importantly, no changes in sexual function or increases in suicidal risk were reported [100]. Additional support for BoNT-A’s favorable safety profile comes from a comparative study with sertraline, in which adverse reactions were observed in 15.38% of BoNT-A-treated patients, significantly lower than the 33.33% reported in the SSRI group [95,115].

To summarize, emerging clinical evidence suggests that BoNT-A may show some effectiveness in the treatment of MDD. Findings from observational and RCTs indicate a reduction in depressive symptoms, with negligible side effects comparable to at least those of sertraline and generally consistent across different populations. Notably, no loss of efficacy has been observed with repeated use. However, controlled trials comparing BoNT-A to common antidepressants are necessary to support or not the literature findings that, at this stage, can be considered interesting, albeit preliminary.

## 4. Discussion

In recent years, BoNT-A has been tested in the treatment of depression, particularly in TRD, with encouraging results, both in real-world [101] and clinical samples with an effect size of d = 0.98, as reported in a meta-analysis published in 2021 [45]. More recently, a systematic review of 21 articles involving a total of 471 patients treated with BoNT-A evaluated its efficacy not only for MDD, but also for bipolar disorder, social anxiety, and BPD, while showing a significant reduction in symptoms related to negative affectivity, generally accompanied by mild and well-tolerated adverse effects [116]. Comparative studies, aimed at better understanding the extent of the effect, showed that the efficacy of this treatment is almost comparable [88], with no sex-related differences [70,101].

Another important aspect to consider is that, once the benefit has worn off, a new administration would seem to allow the restoration of an effect of a magnitude comparable to that of the first one, with the tendency to even improve with the next treatment cycles, providing evidence to support the non-susceptibility to the tolerance mechanism of this treatment [103,108]. As for the dose, the ideal one has not been established yet, although a direct proportionality seems to exist between the depth of the wrinkles on the face and the amount of toxin to be injected to obtain a valid response in terms of efficacy [100,102]. Given the duration of the effect, which is estimated to be about 3 or 4 months, it is believed that this could represent a useful advantage capable of promoting compliance and adherence to the treatment [117].

Interestingly, some scattered data demonstrate that BoNT-A might represent a valid therapeutic strategy even in depressive phases of bipolar disorder [113], as well as in the management of some anxiety disorders [118,119,120] and BPD [121,122].

The mechanisms underlying the antidepressant effects of BoNT-A remain largely unclear; however, the toxin is hypothesized to exert an indirect and direct activity. The indirect effect may be attributable to the facial feedback hypothesis, according to which emotional experience is influenced by facial expression [67]. For this reason, by inhibiting the activity of the corrugator and procerus muscles, mainly involved in the expression of negative affect, BoNT-A might interrupt a feedback loop, reducing proprioceptive and interoceptive afferent signals to brain regions implicated in the processing and regulation of emotions [58]. Neuroimaging evidence supports this theory. while showing that BoNT-A injection can modulate activity in brain regions implicated in emotional regulation, including the hippocampus, amygdala, and medial prefrontal cortex [115].

Regarding the direct effects on the CNS, preclinical studies reported that BoNT-A could undergo retrograde axonal transport from peripheral injection sites to central structures [55]. Therefore, some authors proposed that BoNT-A, by acting on central cholinergic pathways or interneurons through a transsynaptic mechanism, may modulate the release of neurotransmitters, particularly on serotonergic and dopaminergic pathways. However, this theory remains in the realm of hypotheses [115].

Despite the few promising results, significant limitations of the available data should be underlined [123]. Several studies included small sample sizes [95,96,97,99,101,124] and short follow-up times [88,98,100,107], which affect the generalizability of the obtained findings and the ability to assess long-term efficacy. Furthermore, the strong esthetic and psychosocial effects of facial treatments can induce significant placebo responses, introducing significant bias into the study. Heterogeneity in study design in terms of dose administered and facial areas injected further hinders direct comparisons between different studies.

In any case, given the promise in terms of efficacy and rapid onset of action and the low risk profile of BoNT-A, further studies should be conducted to address the mentioned methodological limitations in order to understand whether this innovative treatment involving different branches of medicine can finally obtain regulatory approval for use in the treatment of MDD and TRD.

## 5. Conclusions

The botulin toxin would seem to be a potentially interesting unconventional approach for the treatment of MDD and of TRD in terms of efficacy, rapid onset of therapeutic activity, long duration of action, and few side effects. However, the need for further studies appears evident, given the rather limited number of RCTs, methodological variability, short follow-up durations, and the small sample sizes generally involving only women. It is noteworthy that, although the efficacy is evident, there are still several aspects to be clarified, such as the underlying mechanism of action, the optimal dose, the ideal injection site, and the “real” duration of the effect. For these reasons, the available data should be considered preliminary, and future large-scale, placebo-controlled and active-comparator studies are essential to confirm or refute the antidepressant efficacy of BoNT-A, as well as to clarify optimal dosing, injection sites, and long-term safety.

## Figures and Tables

**Table 1:** The table catalogs 14 “non-conventional” MDD/TRD trials, listing for each the NCT ID, agent/dose/regimen, comparator, design/phase/duration, target population or stratification, planned sample size, primary endpoint/timing, key secondary or mechanistic measures, and recruitment status/dates. The studies cluster into four mechanistic streams: immuno-inflammatory, metabolic/endocrine, rapid glutamatergic/psychedelic modulators (inhaled or IV), and a somatic intervention (whole-body hyperthermia), and range from quadruple-blind Phase 3 RCTs to small open-label feasibility pilots, generally with short follow-ups and limited power, which tempers external validity despite rich biomarker panels.

NCT ID	Agent/Class (Route–Dose–Regimen)	Comparator	Design/Phase/Duration	Population (Key Inclusion/Stratification)	N (Plan)	Primary Endpoint(s) and Timing	Key Secondary/Mechanistic Outcomes	Status and Key Dates
NCT03748446	Xenon-O_2_ (35:65) single inhalation (sub-anesthetic) + TAU	Nitrogen-O_2_ (35:65) + TAU	Randomized, double-blind crossover; Early Phase 1	20 severe depressions: 10 MDD, 10 bipolar depressions (TRD focus)	20	Day-1 improvement on HDRS (6-item) and QIDS-C; repeated acute timepoints	None listed	Recruiting; first posted 20 November 2018; last update 18 May 2025
NCT05357040	Nitrous oxide 25% or 50% (60′ weekly ×4)	Oxygen–air mixture (FiO_2_ ≈ 0.3)	Phase 2, RCT parallel 1:1; nitrous arm split 25% vs. 50%; double-blind (pt/assessor); 7 wk total	Adults with MDD (incl. TRD); outpatient	172	HDRS-21 change over 4 wk	24-h response/remission; POMS; CAT-MH (dep/anx/suicide); S-STS; dose–response; compliance; VAS well-being; AEs	Recruiting; start 30 June 2021; primary compl. 1 October 2025
NCT05415397 (INFLAMED)	Celecoxib 400 mg/day add-on (12 wk)	Placebo add-on	Phase 3, RCT 1:1, parallel, quadruple-blind	DSM-5 MDD with ImmunoMetabolic Depression (IDS AES ≥ 6) + CRP > 1 mg/L; on SSRI/SNRI	140	IDS-SR trajectories (bi-weekly) over 12 wk	IDS response/remission; AES subscore; fatigue, food craving, sleep, anxiety, functioning, pain; pill count; CRP/IL-6/TNF-α/lipids/glucose; AEs	Recruiting; start 28 September 2022; compl. est. July 2025
NCT05558995	Ketogenic diet (20–30 g carbs; 80–100 g protein; fats allowed) 12 wk; adjunct to SSRIs	None (open-label)	Single-arm feasibility; Phase NA; 2-wk induction + 10-wk maintenance	MDD 18–50, partial SSRI responders, residual anhedonia	15	Adherence rate over 12 wk	EEfRT; MADRS; SHAPS; GAD-7; CGI; plasma BDNF and cytokines (TNF-α, IL-1, IL-6, IL-10); extensive safety labs	Recruiting; last update 18 November 2023
NCT05570110	Enoxolone (11β-HSD2 inhibitor) PO; dose NR	Placebo	Randomized, double-blind; biomarker-stratified; Phase NA	MDD; groups split by baseline SBP (median) and urine aldosterone/cortisol; exploratory HRV, sleep, salt taste, CRP, MRI	NR	Differential clinical response by biomarker strata; biomarker change	BP; aldosterone/cortisol; Na^+^/K^+^; HR/HRV; sleep; inflammatory markers; optional MRI/DTI	Recruiting; update 9 April 2024
NCT05570812	Pregnenolone PO ramp 50→500 mg/day (4 wk) then 500 mg/day (4 wk)	Placebo (identical titration)	Phase 2, randomized, parallel, quadruple-blind	PLWH on ART, 18–85 yrs, CES-D ≥ 20; can stay on ADs	120 (90 active/30 plc)	Left insular cortex GABA (MRS) Day14 and Day56 (baseline-adjusted)	CES-D; CD14^+^CD16^+^ monocytes; responder GABA; AEs; dose mods	Recruiting; start 3 March 2023; primary compl. 30 June 2027
NCT05644301 (INSTA-MD)	Minocycline 100 mg BID × 12 wk or Celecoxib 200 mg BID × 12 wk (add-on to TAU)	Placebo + TAU	Phase 3, randomized, parallel, quadruple-blind; hs-CRP stratified (<3/>3 mg/L); 6 arms	DSM-5 MDD, non-remission to adequate AD; physically healthy	240	HDRS-17 change; remission (≤7) at 12 wk	IDS-SR; HDRS response; PSQI; STAI; CORE; MARS; AEs; metabolic markers; cytokines; PBMCs; kynurenine pathway; VEGF, BDNF	Recruiting; start 21 September 2023; compl. est. September 2026
NCT05710887	Nitrous oxide 50% (45′ single session) + TAU in ED	Oxygen–air mixture + TAU	Phase 2, RCT parallel; double-blind (pt/assessor); ED setting; ≤24 h follow-up	18–65, acutely suicidal, non-psychotic MDD in ED	50	CAT-MH change (suicide/dep/anx) within 24 h	Compliance; rapid (30–60′) and sustained response; correlation with lifetime suicide predictors; AEs	Not yet recruiting; start est. 1 October 2025; primary compl. 1 August 2027
NCT05757791	Empagliflozin 10 mg × 14 d → 25 mg × 28 d (6 wk)	None (open-label)	Phase 2, single-group	Adults 18–65, MDD (MADRS ≥ 20), ≤2 failed ADs; no prior SGLT2	16	MADRS change baseline→wk6	C-SSRS; SHAPS	Recruiting; start 17 March 2023; primary compl. est. December 2025
NCT06136546	Infliximab 5 mg/kg IV (single infusion)	Saline IV	Phase 2, randomized, parallel, triple-blind; 2-wk follow-up	MDD, 25–50 yrs, CRP ≥ 3 mg/L; HAMD-17 ≥ 15; stable/off AD ≥ 4 wk	100	Psychomotor speed (Simple RT) and executive function (Choice RT) via TestMyBrain (daily × 2 wk)	HAMD-17; Dimensional Anhedonia Rating Scale; CRP; TNF-α and receptors	Recruiting; start 23 January 2025; primary compl. 31 August 2028
NCT06323785	Whole-body hyperthermia (water-filtered IR)	Sham hyperthermia	RCT, parallel, quadruple-blind; 6 wk; Phase NA	MDD 18–65; HAMD-17 ≥ 14; German-speaking	30	HAMD-17 at 1 wk	BDI; MOS-SF QoL; HAMD-17 at 6 wk	Recruiting; start est. 15 June 2024; primary compl. 1 March 2026
NCT06537921 (CODA)	Minocycline 200 mg/day PO × 8 wk adjunct	None	Single-group, open-label feasibility; 12 wk total	MDD + obesity (BMI ≥ 30) + CRP ≥ 3 mg/L; TRD; MRI-eligible	35	Feasibility: enrolment, adherence, completion of biomarkers/MRI/PROs; effect-size estimates	Blood/saliva biomarkers; MRI baseline and wk 8; questionnaires	Recruiting; start 1 October 2024; compl. est. 1 September 2027
NCT06671977	DMT IV (low and medium bolus+infusion) ± THC comparators	Placebo	Phase 1, randomized crossover; triple-blind; 2 sessions 4 wk apart	Adults 21–65: MDD cohort (moderate–severe, ≥1 inadequate AD) + healthy controls	60	Safety/physiology; MEQ30; PSI; VAS anxiety/dep; CEQ; reinforcing effects; tolerability; EEG	Expectancy/blinding indices; blood assays; NEO; AAQ	Recruiting; start 14 March 2025; compl. est. 1 Decembrer 2027
NCT06698666	Rosuvastatin 10 mg PO daily × 12 wk (± sertraline TAU)	Sertraline (standard care)—details NR	RCT; parallel; convenience sample; Phase NA	Adults 20–45, mild–moderate MDD; MADRS 7–34	144 (72/arm)	MADRS change after 12 wk	Safety AEs; serum cholesterol (baseline and wk 12)	Recruiting; start 2022; last update 21 November 2024

Legend: AD = antidepressant; AES = atypical energy-related symptoms (IDS); AEs = adverse events; BID = twice daily; CAT-MH = Computerized Adaptive Testing–Mental Health; CGI = Clinical Global Impression; CRP = C-reactive protein; DTI = diffusion tensor imaging; ED = emergency department; EEfRT = Effort Expenditure for Rewards Task; HAMD/HDRS = Hamilton Depression Rating Scale; HRV = heart rate variability; IDS-SR = Inventory of Depressive Symptomatology–Self Report; IMD = ImmunoMetabolic Depression; MRS = magnetic resonance spectroscopy; PBMC = peripheral blood mononuclear cells; POMS = Profile of Mood States; PSQI = Pittsburgh Sleep Quality Index; QIDS-C = Quick Inventory of Depressive Symptomatology–Clinician; SBP = systolic blood pressure; SDS = Sheehan Disability Scale; SHAPS = Snaith-Hamilton Pleasure Scale; S-STS = Sheehan Suicidality Tracking Scale; TAU = treatment as usual; TRD = treatment-resistant depression; VAS = visual analog scale; wk = week.

**Table 2:** Caption. Preclinical evidence on botulinum neurotoxins: key experimental studies and their effects on neurotransmission and behavior.

Authors and Year of Publication	Type of Study	Study Sample	Type of Intervention	Comparison	Evaluation Tools	Results
Burgen et al. (1949) [54].	Experimental, observational physiology.	Isolated neuromuscular junction preparations from rats.	Application of botulinum toxin to muscle-nerve preparations.	N.A.	Electrophysiology (muscle contraction amplitude).	Botulinum toxin blocks acetylcholine release at the neuromuscular junction, leading to progressive paralysis.
Blasi et al. (1993) [38].	Experimental, biochemical comparative.	Cultured neurons and recombinant proteins.	Exposure of neurons to BoNT/C1.	Cleaved vs. intact syntaxin.	Protein cleavage assays, neurotransmitter release quantification.	BoNT/C1 specifically cleaves syntaxin (HPC-1), blocking synaptic vesicle exocytosis and thus neurotransmitter release.
Sikorra et al. (2008) [39].	Experimental, biochemical comparative.	Recombinant synaptobrevin/VAMPs.	Exposure to different VAMP-cleaving clostridial.	Comparisons among BoNT serotypes.	Site-directed mutagenesis, proteolytic cleavage assays, structural modeling.	Identification of specific aminoacidic residues critical for VAMP recognition and cleavage by BoNT serotypes, explaining substrate selectivity.
Restani et al. (2012) [55].	Experimental, in vivo observational.	Rodents (mice/rats).	Peripheral BoNT/A injection into retina or nerve terminals.	Injected vs. non-injected controls.	Immunohistochemistry, electrophysiology, synaptic transmission assays.	BoNT/A undergoes retrograde axonal transport and is trans-synaptically transferred, impairing neurotransmission at second-order neurons.
Caleo et al. (2018) [49].	Experimental, in vivo observational.	Rodents (mice).	Peripheral BoNT/A injection.	Injected vs. control groups.	Immunohistochemistry, neuronal activity mapping, synaptic staining.	BoNT/A crosses synapses trans-synaptically, altering neurotransmission in central cholinergic boutons distant from injection site.
Li et al. (2019) [52].	Experimental, in vivo comparative.	Mice.	Single facial injection of BoNT/A vs. saline control.	BoNT/A vs. saline control.	Behavioral tests (forced swim test, tail suspension test), neurochemical assays (5-HT, BDNF, ERK/CREB activation).	BoNT/A-treated mice showed reduced depressive-like behavior, increased serotonin levels, and activation of BDNF/ERK/CREB pathways, suggesting antidepressant potential.
Ni et al. (2023) [53].	Experimental, in vivo comparative.	Mice.	Retrograde facial BoNT/A injection.	BoNT/A vs. controls; chemogenetic silencing vs. intact pathways.	Behavioral tests, neuronal tracing, chemogenetic manipulation, immunohistochemistry.	Mapped neural circuits mediating BoNT/A’s antidepressant effect, identifying serotonergic projections as critical; silencing these pathways abolished behavioral improvement.

Legend: 5-HT = 5-Hydroxytryptamine (Serotonin); BDNF = Brain-Derived Neurotrophic Factor; BoNT = Botulinum Neurotoxin; BoNT/A = Botulinum Neurotoxin serotype A; BoNT/C1 = Botulinum Neurotoxin serotype C, subtype 1; CREB = cAMP Response Element-Binding Protein; ERK = Extracellular signal-Regulated Kinase; HPC-1 = Human Pancreatic Carcinoma-1 (syntaxin isoform, neuronal); SNAP-25 = Synaptosomal-Associated Protein of 25 kDa; VAMP = Vesicle-Associated Membrane Protein.

## References

[1] Erchinger V.J., Ersland L., Aukland S.M., Abbott C.C., Oltedal L. (2021). Magnetic resonance spectroscopy in depressed subjects treated with electroconvulsive therapy—A systematic review of literature. Front. Psychiatry.

[2] Monroe S.M., Harkness K.L. (2022). Major depression and its recurrences: Life course matters. Annu. Rev. Clin. Psychol..

[3] GBD 2021 Diseases and Injuries Collaborators (2024). Global incidence, prevalence, years lived with disability (YLDs), disability-adjusted life-years (DALYs), and healthy life expectancy (HALE) for 371 diseases and injuries in 204 countries and territories and 811 subnational locations, 1990–2021: A systematic analysis for the Global Burden of Disease Study 2021. Lancet.

[4] Otte C., Gold S.M., Penninx B.W., Pariante C.M., Etkin A., Fava M., Mohr D.C., Schatzberg A.F. (2016). Major depressive disorder. Nat. Rev. Dis. Primers.

[5] Li W., Zhao Z., Chen D., Peng Y., Lu Z. (2022). Prevalence and associated factors of depression and anxiety symptoms among college students: A systematic review and meta-analysis. J. Child Psychol. Psychiatry.

[6] Ohayon M.M., Schatzberg A.F. (2003). Using chronic pain to predict depressive morbidity in the general population. Arch. Gen. Psychiatry.

[7] Liu W., Ge T., Leng Y., Pan Z., Fan J., Yang W., Cui R. (2017). The role of neural plasticity in depression: From hippocampus to prefrontal cortex. Neural Plast..

[8] Wohleb E.S., Franklin T., Iwata M., Duman R.S. (2016). Integrating neuroimmune systems in the neurobiology of depression. Nat. Rev. Neurosci..

[9] Dean J., Keshavan M. (2017). The neurobiology of depression: An Integrated View. Asian J. Psychiatry.

[10] Jiang Y., Zou D., Li Y., Gu S., Dong J., Ma X., Xu S., Wang F., Huang J.H. (2022). Monoamine neurotransmitters control basic emotions and affect major depressive disorders. Pharmaceuticals.

[11] Shao X., Zhu G. (2020). Associations among monoamine neurotransmitter pathways, personality traits, and major depressive disorder. Front. Psychiatry.

[12] Liu X., Liu X., Wang Y., Zeng B., Zhu B., Dai F. (2023). Association between depression and oxidative balance score: National Health and Nutrition Examination Survey (NHANES) 2005–2018. J. Affect. Disord..

[13] Karabin T., Biala G., Kruk-Slomka M. (2023). The monoamine theory of depression as a target to effective pharmacotherapy. Curr. Issues Pharm. Med. Sci..

[14] Schmaal L., Veltman D.J., van Erp T.G.M., Sämann P.G., Frodl T., Jahanshad N., Loehrer E., Tiemeier H., Hofman A., Niessen W.J. (2016). Subcortical brain alterations in major depressive disorder: Findings from the ENIGMA major depressive disorder working group. Mol. Psychiatry.

[15] Schmaal L., Pozzi E., Ho T.C., Van Velzen L.S., Veer I.M., Opel N., Van Someren E.J., Han L.K., Aftanas L., Aleman A. (2020). ENIGMA MDD: Seven years of global neuroimaging studies of major depression through worldwide data sharing. Transl. Psychiatry.

[16] Van Velzen L.S., Kelly S., Isaev D., Aleman A., Aftanas L.I., Bauer J., Baune B.T., Brak I.V., Carballedo A., Connolly C.G. (2020). White matter disturbances in major depressive disorder: A coordinated analysis across 20 International cohorts in the ENIGMA MDD working group. Mol. Psychiatry.

[17] Strawbridge R., Young A.H., Cleare A.J. (2017). Biomarkers for depression: Recent insights, current challenges and future prospects. Neuropsychiatr. Dis. Treat..

[18] Jiao Y., Zhao K., Wei X., Carlisle N.B., Keller C.J., Oathes D.J., Fonzo G.A., Zhang Y. (2025). Deep graph learning of multimodal brain networks defines treatment-predictive signatures in major depression. Mol. Psychiatry.

[19] Castrén E., Monteggia L.M. (2021). Brain-derived neurotrophic factor signaling in depression and antidepressant action. Biol. Psychiatry.

[20] Rush A.J., Trivedi M.H., Wisniewski S.R., Nierenberg A.A., Stewart J.W., Warden D., Niederehe G., Thase M.E., Lavori P.W., Lebowitz B.D. (2006). Acute and longer-term outcomes in depressed outpatients requiring one or several treatment steps: A STAR*D Report. Am. J. Psychiatry.

[21] Gaynes B.N., Lux L., Gartlehner G., Asher G., Forman-Hoffman V., Green J., Boland E., Weber R.P., Randolph C., Bann C. (2020). Defining treatment-resistant depression. Depress. Anxiety.

[22] Malhi G.S., Byrow Y. (2016). Is treatment-resistant depression a useful concept?. BMJ Ment. Health.

[23] Zhdanava M., Pilon D., Ghelerter I., Chow W., Joshi K., Lefebvre P., Sheehan J.J. (2021). The prevalence and national burden of treatment-resistant depression and major depressive disorder in the United States. J. Clin. Psychiatry.

[24] Fava M., Freeman M.P., Flynn M., Judge H., Hoeppner B.B., Cusin C., Ionescu D.F., Mathew S.J., Chang L.C., Iosifescu D.V. (2020). Double-blind, placebo-controlled, dose-ranging trial of intravenous ketamine as adjunctive therapy in treatment-resistant depression (TRD). Mol. Psychiatry.

[25] Wiesinger T., Kremer S., Bschor T., Baethge C. (2023). Antidepressants and quality of life in patients with major depressive disorder—Systematic review and meta-analysis of double-blind, placebo-controlled RCTs. Acta Psychiatry Scand..

[26] Saelens J., Gramser A., Watzal V., Zarate C.A., Lanzenberger R., Kraus C. (2025). Relative effectiveness of antidepressant treatments in treatment-resistant depression: A systematic review and network meta-analysis of randomized controlled trials. Neuropsychopharmacology.

[27] Gelenberg A.J., Freeman M.P., Markowitz J.C., Rosenbaum J.F., Thase M.E., Trivedi M.H., Van Rhoads R.S. (2010). American Psychiatric Association practice guideline for the treatment of patients with Major Depressive Disorder. Am. J. Psychiatry.

[28] National Institute for Health and Care Excellence (NICE) (2022). Depression in Adults: Treatment and Management.

[29] Marazziti D. (2025). Capitolo 3. Depressione Resistente. Psicofarmacoterapia Clinica.

[30] Frevert J. (2015). Pharmaceutical, biological, and clinical properties of botulinum neurotoxin type A products. Drugs RD.

[31] Frevert J., Dressler D. (2016). Clinical Relevance of Immunoresistance to Botulinum Therapy. Botulinum Toxin Therapy Manual for Dystonia and Spasticity.

[32] Albrecht P., Jansen A., Lee J.-I., Moll M., Ringelstein M., Rosenthal D., Bigalke H., Aktas O., Hartung H.-P., Hefter H. (2019). High prevalence of neutralizing antibodies after long-term botulinum neurotoxin therapy. Neurology.

[33] Dessy L.A., Fallico N., Mazzocchi M., Scuderi N. (2011). Botulinum toxin for glabellar lines: A review of the efficacy and safety of currently available products. Am. J. Clin. Dermatol..

[34] Souery D., Papakostas G.I., Trivedi M.H. (2006). Treatment-resistant depression. J. Clin. Psychiatry.

[35] Wijeratne C., Sachdev P. (2008). Treatment-resistant depression: Critique of current approaches. Aust. N. Z. J. Psychiatry.

[36] Aoki K.R. (2005). Review of a proposed mechanism for the antinociceptive action of botulinum toxin type A. Neurotoxicology.

[37] Aoki K.R., Guyer B. (2001). Botulinum toxin type A and other botulinum toxin serotypes: A comparative review of biochemical and pharmacological actions. Euro J. Neurol..

[38] Blasi J., Chapman E.R., Yamasaki S., Binz T., Niemann H., Jahn R. (1993). Botulinum neurotoxin C1 blocks neurotransmitter release by means of cleaving HPC-1/syntaxin. EMBO J..

[39] Sikorra S., Henke T., Galli T., Binz T. (2008). Substrate recognition mechanism of VAMP/synaptobrevin-cleaving clostridial neurotoxins. J. Biol. Chem..

[40] Carr W.W., Jain N., Sublett J.W. (2021). Immunogenicity of botulinum toxin formulations: Potential therapeutic implications. Adv. Ther..

[41] Ayoub N. (2025). Botulinum toxin therapy: A comprehensive review on clinical and pharmacological insights. J. Clin. Med..

[42] Mazzocchio R., Caleo M. (2015). More than at the neuromuscular synapse: Actions of botulinum neurotoxin A in the central nervous system. Neuroscientist.

[43] Pirazzini M., Rossetto O., Eleopra R., Montecucco C. (2017). Botulinum neurotoxins: Biology, pharmacology, and toxicology. Pharmacol. Rev..

[44] Caleo M., Restani L. (2018). Direct central nervous system effects of botulinum neurotoxin. Toxicon.

[45] Schulze J., Neumann I., Magid M., Finzi E., Sinke C., Wollmer M.A., Krüger T.H.C. (2021). Botulinum toxin for the management of depression: An updated review of the evidence and meta-analysis. J. Psychiatr. Res..

[46] Qian H., Shao F., Lenahan C., Shao A., Li Y. (2020). Efficacy and safety of botulinum toxin vs. placebo in depression: A systematic review and meta-analysis of randomized controlled trials. Front. Psychiatry.

[47] Crowley J.S., Silverstein M.L., Reghunathan M., Gosman A.A. (2022). Glabellar botulinum toxin injection improves depression scores: A systematic review and meta-analysis. Plast. Reconstr. Surg..

[48] Arnone D., Galadari H., Rodgers C.J., Östlundh L., Aziz K.A., Stip E., Young A.H. (2021). Efficacy of onabotulinumtoxinA in the treatment of unipolar major depression: Systematic review, meta-analysis and meta-regression analyses of double-blind randomised controlled trials. J. Psychopharmacol..

[49] Caleo M., Spinelli M., Colosimo F., Matak I., Rossetto O., Lackovic Z., Restani L. (2018). Transynaptic action of botulinum neurotoxin type A at central cholinergic boutons. J. Neurosci..

[50] Hallett M. (2018). Mechanism of action of botulinum neurotoxin: Unexpected consequences. Toxicon.

[51] Makunts T., Wollmer M.A., Abagyan R. (2020). Postmarketing safety surveillance data reveals antidepressant effects of botulinum toxin across various indications and injection sites. Sci. Rep..

[52] Li Y., Liu J., Liu X., Su C.-J., Zhang Q.-L., Wang Z.-H., Cao L.-F., Guo X.-Y., Huang Y., Luo W. (2019). Antidepressant-like action of single facial injection of botulinum neurotoxin A is associated with augmented 5-HT Levels and BDNF/ERK/CREB Pathways in Mouse Brain. Neurosci. Bull..

[53] Ni L., Chen H., Xu X., Sun D., Cai H., Wang L., Tang Q., Hao Y., Cao S., Hu X. (2023). Neurocircuitry underlying the antidepressant effect of retrograde facial botulinum toxin in mice. Cell Biosci..

[54] Burgen A.S.V., Dickens F., Zatman L.J. (1949). The action of botulinum toxin on the neuro-muscular junction. J. Physiol..

[55] Restani L., Novelli E., Bottari D., Leone P., Barone I., Galli-Resta L., Strettoi E., Caleo M. (2012). Botulinum neurotoxin A impairs neurotransmission following retrograde transynaptic transport. Traffic.

[56] Ekman P. (2007). The directed facial action task: Emotional responses without appraisal. Handbook of Emotion Elicitation and Assessment.

[57] Schwartz G.E., Fair P.L., Salt P., Mandel M.R., Klerman G.L. (1976). Facial muscle patterning to affective imagery in depressed and nondepressed subjects. Science.

[58] Rodríguez-Cerdeira C., Eckhardt W. (2024). Depression treatment: Is there a role for botulinum toxin type A?. Microorganisms.

[59] Heckmann M., Teichmann B., Schröder U., Sprengelmeyer R., Ceballos-Baumann A.O. (2003). Pharmacologic denervation of frown muscles enhances baseline expression of happiness and decreases baseline expression of anger, sadness, and fear. J. Am. Acad. Dermatol..

[60] Wollmer M.A., Magid M., Kruger T.H.C., Finzi E., Whitcup S.M., Hallett M. (2021). The use of botulinum toxin for treatment of depression. Botulinum Toxin Therapy.

[61] Darwin C. (1872). The Expression of the Emotions in Man and Animals.

[62] Strack F., Martin L.L., Stepper S. (1988). Inhibiting and facilitating conditions of the human smile: A nonobtrusive test of the facial feedback hypothesis. J. Personal. Soc. Psychol..

[63] Lin J.-G., Kotha P., Chen Y.-H. (2022). Understandings of acupuncture application and mechanisms. Am. J. Transl. Res..

[64] Wollmer M.A., Kalak N., Jung S., DeBoer C., Magid M., Reichenberg J.S., Brand S., Holsboer-Trachsler E., Kruger T.H.C. (2014). Agitation predicts response of depression to botulinum toxin treatment in a randomized controlled trial. Front. Psychiatry.

[65] Brennan C. (2016). Botulinum toxin type-A (BoNT-A) injections of the corrugator muscles for aesthetics and depression?. Plast. Surg. Nurs..

[66] Finzi E. (2013). The Face of Emotion: How Botox Affects Our Mood and Relationships.

[67] Finzi E., Rosenthal N.E. (2016). Emotional proprioception: Treatment of depression with afferent facial feedback. J. Psychiatr. Res..

[68] Al Abdulmohsen T., Kruger T.H.C. (2011). The contribution of muscular and auditory pathologies to the symptomatology of autism. Med. Hypotheses.

[69] Davis J.I., Senghas A., Brandt F., Ochsner K.N. (2010). The effects of BOTOX injections on emotional experience. Emotion.

[70] Lewis M.B., Bowler P.J. (2009). Botulinum toxin cosmetic therapy correlates with a more positive mood. J. Cosmet. Dermatol..

[71] Sommer B., Zschocke I., Bergfeld D., Sattler G., Augustin M. (2003). Satisfaction of patients after treatment with botulinum toxin for dynamic facial lines. Dermatol. Surg..

[72] Sykianakis D., Stratigos A., Chatziioannou A., Christodoulou C. (2022). Botulinum toxin type A treatment is associated with improved social and psychological behavior: A retrospective study. J. Cosmet. Dermatol..

[73] Baumeister J.-C., Papa G., Foroni F. (2016). Deeper than Skin Deep—The Effect of Botulinum Toxin-A on Emotion Processing. Toxicon.

[74] Bulnes L.C., Mariën P., Vandekerckhove M., Cleeremans A. (2019). The effects of botulinum toxin on the detection of gradual changes in facial emotion. Sci. Rep..

[75] Havas D.A., Glenberg A.M., Gutowski K.A., Lucarelli M.J., Davidson R.J. (2010). Cosmetic use of botulinum toxin-A affects processing of emotional language. Psychol. Sci..

[76] Hennenlotter A., Dresel C., Castrop F., Ceballos-Baumann A.O., Wohlschläger A.M., Haslinger B. (2009). The link between facial feedback and neural activity within central circuitries of emotion—New insights from botulinum toxin–induced denervation of frown muscles. Cereb. Cortex.

[77] Kim M.J., Neta M., Davis F.C., Ruberry E.J., Dinescu D., Heatherton T.F., Stotland M.A., Whalen P.J. (2014). Botulinum toxin-induced facial muscle paralysis affects amygdala responses to the perception of emotional expressions: Preliminary findings from an A-B-A design. Biol. Mood Anxiety Disord..

[78] Shin L.M., Liberzon I. (2010). The neurocircuitry of fear, stress, and anxiety disorders. Neuropsychopharmacology.

[79] Matsuo K., Ban R., Hama Y., Yuzuriha S. (2015). Eyelid opening with trigeminal proprioceptive activation regulates a brainstem arousal mechanism. PLoS ONE.

[80] Finzi E. (2023). Botulinum toxin treatment for depression: A new paradigm for psychiatry. Toxins.

[81] Wollmer M.A., Magid M., Kruger T.H.C., Finzi E. (2022). Treatment of depression with botulinum toxin. Toxins.

[82] Myers B. (2017). Corticolimbic regulation of cardiovascular responses to stress. Physiol. Behav..

[83] Krohn F., Novello M., van der Giessen R.S., De Zeeuw C.I., Pel J.J.M., Bosman L.W.J. (2023). The integrated brain network that controls respiration. eLife.

[84] Šimić G., Tkalčić M., Vukić V., Mulc D., Španić E., Šagud M., Olucha-Bordonau F.E., Vukšić M., Hof P.R. (2021). Understanding emotions: Origins and roles of the amygdala. Biomolecules.

[85] Lamotte G., Shouman K., Benarroch E.E. (2021). Stress and central autonomic network. Auton. Neurosci..

[86] Quadt L., Critchley H., Nagai Y. (2022). Cognition, emotion, and the central autonomic network. Auton. Neurosci..

[87] Zhang X., Ge T.T., Yin G., Cui R., Zhao G., Yang W. (2018). Stress-induced functional alterations in amygdala: Implications for neuropsychiatric diseases. Front. Neurosci..

[88] Zhang Q., Wu W., Fan Y., Li Y., Liu J., Xu Y., Jiang C., Tang Z., Cao C., Liu T. (2021). The safety and efficacy of botulinum toxin A on the treatment of depression. Brain Behav..

[89] Bang J.Y., Zhao J., Rahman M., St-Cyr S., McGowan P.O., Kim J.C. (2022). Hippocampus-anterior hypothalamic circuit modulates stress-induced endocrine and behavioral response. Front. Neural Circuits.

[90] Keynan J.N., Meir-Hasson Y., Gilam G., Cohen A., Jackont G., Kinreich S., Ikar L., Or-Borichev A., Etkin A., Gyurak A. (2016). Limbic activity modulation guided by functional magnetic resonance imaging–inspired electroencephalography improves implicit emotion regulation. Biol. Psychiatry.

[91] Barreiros A.R., Almeida I., Baía B.C., Castelo-Branco M. (2019). Amygdala modulation during emotion regulation training with fMRI-cased neurofeedback. Front. Hum. Neurosci..

[92] Jentsch V.L., Merz C.J., Wolf O.T. (2019). Restoring emotional stability: Cortisol effects on the neural network of cognitive emotion regulation. Behav. Brain Res..

[93] Parsaik A.K., Mascarenhas S.S., Hashmi A., Prokop L.J., John V., Okusaga O., Singh B. (2016). Role of botulinum toxin in depression. J. Psychiatr. Pract..

[94] Magid M., Finzi E., Kruger T.H.C., Robertson H.T., Keeling B.H., Jung S., Reichenberg J.S., Rosenthal N.E., Wollmer M.A. (2015). Treating depression with botulinum toxin: A pooled analysis of randomized controlled trials. Pharmacopsychiatry.

[95] Hexsel D., Hexsel C., Siega C., Schilling-Souza J., Rotta F.T., Rodrigues T.C. (2013). Fields of effects of 2 commercial preparations of botulinum toxin type A at equal labeled unit doses: A double-blind randomized trial. JAMA Dermatol..

[96] Finzi E., Wasserman E. (2006). Treatment of depression with botulinum toxin A: A case series. Dermatol. Surg..

[97] Wollmer M.A., de Boer C., Kalak N., Beck J., Götz T., Schmidt T., Hodzic M., Bayer U., Kollmann T., Kollewe K. (2012). Facing depression with botulinum toxin: A randomized controlled trial. J. Psychiatr. Res..

[98] Finzi E., Rosenthal N.E. (2014). Treatment of depression with onabotulinumtoxinA: A randomized, double-blind, placebo controlled trial. J. Psychiatr. Res..

[99] Magid M., Reichenberg J.S., Poth P.E., Robertson H.T., LaViolette A.K., Kruger T.H.C., Wollmer M.A. (2014). Treatment of major depressive disorder using botulinum toxin A: A 24-week randomized, double-blind, placebo-controlled study. J. Clin. Psychiatry.

[100] Brin M.F., Durgam S., Lum A., James L., Liu J., Thase M.E., Szegedi A. (2020). OnabotulinumtoxinA for the treatment of major depressive disorder: A phase 2 randomized, double-blind, placebo-controlled trial in adult females. Int. Clin. Psychopharmacol..

[101] Chugh S., Chhabria A., Jung S., Kruger T.H.C., Wollmer M.A. (2018). Botulinum toxin as a treatment for depression in a real-world setting. J. Psychiatr. Pract..

[102] Shu H., Shen T., Deng W., Cao J., Xu Y., Liu J., Zhou X., Luo W.F. (2024). Comparative effectiveness of two different doses of botulinum toxin A for the treatment of mild to moderate depression. J. Affect. Disord..

[103] Cristel R.T., Gandhi N.D., Issa T.Z., Kola E., Demesh D., Dayan S.H. (2021). A Randomized, single-blind, crossover study evaluating the impact of onabotulinumtoxinA treatment on mood and appearance during the COVID-19 pandemic. Aesthet. Surg. J..

[104] Klassen A.F., Cano S.J., Scott A., Snell L., Pusic A.L. (2010). Measuring patient-reported outcomes in facial aesthetic patients: Development of the FACE-Q. Facial Plast. Surg..

[105] Lyubomirsky S., Lepper H.S. (1999). A measure of subjective happiness: Preliminary reliability and construct validation. Soc. Indic. Res..

[106] Feng X.-Y., Shen T.-T., Wu Q.-C., Wang J., Ni P., Liu J., Zhou X.-P., Hu H., Luo W.-F. (2024). A novel approach to treating post-stroke depression: Administration of botulinum toxin A via local facial injection. Front. Neurol..

[107] Li Y., Zhu T., Shen T., Wu W., Cao J., Sun J., Liu J., Zhou X., Jiang C., Tang Z. (2022). Botulinum toxin A (BoNT/A) for the treatment of depression: A randomized, double-blind, placebo, controlled trial in China. J. Affect. Disord..

[108] Lehnert F., Neumann I., Krüger T.H.C., Wollmer M.A. (2023). Botulinum toxin therapy for psychiatric disorders in clinical practice: A retrospective case study. Toxins.

[109] Kruger T.H.C., Magid M., Wollmer M.A. (2016). Can botulinum toxin help patients with borderline personality disorder?. Am. J. Psychiatry.

[110] Kruger T.H.C., Schulze J., Bechinie A., Neumann I., Jung S., Sperling C., Engel J., Müller A., Kneer J., Kahl K.G. (2022). Neuronal effects of glabellar botulinum toxin injections using a valenced inhibition task in borderline personality disorder. Sci. Rep..

[111] Ceolato-Martin C., Chevallier-Collins C., Clément J.-P., Charles E., Lacroix A., Ranoux D. (2024). OnabotulinumtoxinA in resistant depression: A randomized trial comparing two facial injection sites (OnaDEP Study). Depress. Anxiety.

[112] Guvenc U. (2023). Botulinum toxin and its effect on depression. EJMI.

[113] Finzi E., Kels L., Axelowitz J., Shaver B., Eberlein C., Krueger T.H., Wollmer M.A. (2018). Botulinum toxin therapy of bipolar depression: A case series. J. Psychiatr. Res..

[114] Farooqui A.A., Fulkerson J.M., El-Mallakh R.S. (2023). Use of botulinum toxin A for depression symptoms in patients with treatment-resistant bipolar illness: A case series. Bipolar Disord..

[115] Li Y., Liu T., Luo W. (2021). Botulinum neurotoxin therapy for depression: Therapeutic mechanisms and future perspective. Front. Psychiatry.

[116] Demchenko I., Swiderski A., Liu H., Jung H., Lou W., Bhat V. (2024). Botulinum toxin injections for psychiatric disorders: A systematic review of the clinical trial landscape. Toxins.

[117] Naumann M., Jankovic J. (2004). Safety of botulinum toxin type A: A systematic review and meta-analysis. Curr. Med. Res. Opin..

[118] Connor K.M., Cook J.L., Davidson J.R.T. (2006). Botulinum toxin treatment of social anxiety disorder with hyperhidrosis: A placebo-controlled double-blind trial. J. Clin. Psychiatry.

[119] Dong H., Fan S., Luo Y., Peng B. (2019). Botulinum toxin relieves anxiety and depression in patients with hemifacial spasm and blepharospasm. Neuropsychiatr. Dis. Treat..

[120] Wang Y., Yang X., Ji X., Liu M., Zhou C. (2023). Clinical efficacy of escitalopram combined with botulinum toxin A in patients with generalized anxiety disorder and comorbid headache. Psychopharmacology.

[121] Wollmer M.A., Neumann I., Jung S., Bechinie A., Herrmann J., Müller A., Wohlmuth P., Fournier-Kaiser L., Sperling C., Peters L. (2022). Clinical effects of glabellar botulinum toxin injections on borderline personality disorder: A randomized controlled trial. J. Psychopharmacol..

[122] Schulze J., Sinke C., Neumann I., Wollmer M.A., Kruger T.H.C. (2024). Effects of glabellar botulinum toxin injections on resting-state functional connectivity in borderline personality disorder. Eur. Arch. Psychiatry Clin. Neurosci..

[123] Stearns T.P., Shad M.U., Guzman G.C. (2018). Glabellar botulinum toxin injections in major depressive disorder: A critical review. Prim. Care Companion CNS Disord..

[124] Zamanian A., Ghanbari Jolfaei A., Mehran G., Azizian Z. (2017). Efficacy of botox versus placebo for treatment of patients with major depression. Iran. J. Public Health.

